# Microbiota restoration therapies for recurrent *Clostridioides difficile* infection reach an important new milestone

**DOI:** 10.1177/17562848241253089

**Published:** 2024-05-24

**Authors:** Herbert L. DuPont, Andrew W. DuPont, Glenn S. Tillotson

**Affiliations:** Infectious Diseases and Epidemiology, Department of Epidemiology, University of Texas School of Public Health, 1200 Pressler Street, Houston, TX 77030, USA; Department of Internal Medicine, University of Texas McGovern Medical School, Houston, TX, USA; Department of Medicine, Baylor College of Medicine, Houston, TX, USA; Kelsey Research Foundation, Houston, TX, USA; Division of Gastroenterology, University of Texas Health Science Center at Houston, Houston, TX, USA; GST Micro LLC, North, VA, USA

**Keywords:** *Clostridioides difficile*, fecal microbiota transplantation, live biotherapeutic products, REBYOTA™, VOWST

## Abstract

Microbiota restoration therapy has become a standard treatment for recurrent *Clostridioides difficile* infection (rCDI). In this article, we review the studies supporting the licensure of two live biotherapeutic products (LBPs) designed to prevent rCDI and to provide clinicians with a perspective on their differences. PubMed was reviewed on 1 October 2023, for all papers published concerning the current Food and Drug Administration allowance of the use of fecal microbiota transplantation (FMT) and the studies that led to the licensure of RBX2660 (REBYOTA™), generic name, fecal microbiota, live-jslm, and SER-109 (VOWST™), generic name, fecal microbiota spores, live-brpk. OpenBiome continues to produce fecal products for patients with rCDI at their treatment sites, and the American Gastroenterology Association has a National Registry focused on long-term safety of administering fecal microbiota products. The science behind the licensing of fecal microbiota, live-jslm, a consortium of fecal anaerobes found in stool augmented with strains of Bacteroidetes and fecal microbiota spores, live-brpk, a mixture of 50 species of purified Firmicutes spores is reviewed. Both products appear to be safe in clinical trials and effective in reducing rCDI episodes by mechanisms established for FMT, including normalization of α- and β-diversity of the microbiome and by increasing fecal secondary bile acids. The different makeup of the two LBPs suggests that rCDI responds to a variety of engrafting microbiota which explains why nearly all donors in FMT of rCDI are generally effective. Fecal microbiota, live-jslm has also been shown to successfully treat rCDI in elderly patients with advanced comorbidities. With the licensure of two novel LBPs, we are entering a new phase of microbiota replacement therapy. Having standardized manufacturing and proper monitoring of products, harnessing the microbiome to control and prevent disease has a new beginning.

## Plain language summary

Licensure of two new live biotherapeutic products to treat recurrent *C difficile* infection is changing the landscape for treatment of this common and often serious infection

Microbiota replacement therapy is the most effective way to prevent multiple recurrences of *C difficile* infection. The article discusses where fecal microbiota transplantation is available in North America. The major focus is on two recently licensed live biotherapeutic products, RBX2660 (REBYOTA), generic name fecal microbiota, live-jslm and SER-109 (VOWST), generic fecal microbiota spores, live-brpk, manufactured under standardized methods which should be safer and more standardized in response. The article compares the new LBPs for safety, effectiveness, cost to help clinicians make decisions. The licensure and availability of two safe and effective standardized and regulated biotherapies, fecal microbiota, live-jslm and fecal microbiota spores, live-brpk, for preventing rCDI is a critical advance in medical management. Both treatments were shown to cure rCDI, to normalize the microbiome of the treated patients by reducing proportions of proinflammatory Enterobacteriaceae and increasing the α- and β-diversity of the microbiome, and to convert primary bile acids to *C. difficile*-inhibiting secondary bile acids in fecal samples. Both products included follow-up studies show durable cure without important short-term adverse events. The two recently licensed LBP differ in a number of ways. Fecal microbiota, live-jslm is a broad consortium of microbiota expected in a healthy donor fecal samples, including all the major phyla including Firmicutes. It is augmented with strains of Bacteroidetes, while fecal microbiota spores, live-brpk is ethanol washed spores exclusively within the phylum of Firmicutes. The fact that both products are effective in preventing rCDI support the idea that bacterial restoration in rCDI can be achieved by transplantation of a variety of different microbiota. This is seen in FMT for rCDI where it is generally accepted that all healthy adults are suitable donors and large number of donors can be included unscreened for microbiome diversity in a stool bank such as OpenBiome. When treating conditions other than CDI, the specific makeup of an LBP may need to be adjusted. One way around the unique microbiome requirements of non-CDI illnesses with dysbiosis is to administer FMT product derived from multiple donors. Evidence developed and presented here indicate that the two new LBPs are effective in treating rCDI, although head-to-head comparisons have not been carried out. fecal microbiota, live-jslm is a more traditional microbiome restoration product employing a full range of microbiota. fecal microbiota spores, live-brpk is novel in design and is based on the selection of Firmicutes spores with a narrower range of bioactivity. The future of microbiota-therapy has gotten brighter with the licensure of fecal microbiota, live-jslm and fecal microbiota spores, live-brpk.

## Introduction

The rise in the use of antibiotics with anaerobic activity has fueled the major increase in the frequency of *Clostridioides difficile* infection (CDI).^1^ The most common complication of CDI is recurrence which is caused by spores of *C. difficile* in the gut germinating in the presence of primary bile salts in the setting of dysbiosis,^2^ processes that are reversed by microbiota transplantation.^3^ It has been estimated that the number of multiple recurrences of CDI in the United States each year is approximately 48,000 and that microbiota restoration therapy could prevent 32,000 *C. difficile* recurrences.^4^

Fecal microbiota transplantation (FMT) has become an important therapy for recurrent CDI (rCDI) refractory to other treatments, recognized by the U.S. American College of Gastroenterology^5^ and the British Society of Gastroenterology and Healthcare Infection Society,^6^ although it is considered a research approach used without licensed approval.

Recently, the field of microbiota restoration therapy has moved forward significantly with the U.S. Food and Drug Administration (FDA) approvals of two live biotherapeutic agents (live biotherapeutic products, LBP). These pioneering advances will rapidly change the landscape in preventing the public health burden of rCDI.

This review describes the short history of widespread use of FMT in the United States for refractory rCDI and the currently available programs for the microbiologic treatment of rCDI and then concentrates on the science behind the two newly licensed products, REBYOTA™, Ferring Pharmaceuticals and VOWST™, Seres Therapeutics discussing their anticipated value in dealing with the growing problem of rCDI. The objective of this review is to serve as a guide for physicians and patients considering treatment for rCDI. In the following, REBYOTA will be referred to as fecal microbiota, live-jslm, and VOWST as fecal microbiota spores, live-brpk.

On 18 July 2013, the FDA allowed the use of FMT for Refractory rCDI after holding a public workshop on 2 and 3 May 2013, co-sponsored by the National Institutes of Health (NIH) and the U.S. FDA’s Center for Biologics Evaluation and Research. The FDA published a new policy to exercise enforcement discretion regarding the broad use of unlicensed FMT without requiring an investigational new drug (IND) application for the treatment of patients with rCDI unresponsive to other treatments.^7^ Since then, the Agency has issued multiple guidance documents on FMT, with the most recent published on 29 November 2022,^8^ again supporting the use of FMT without an IND for refractory CDI. Thorough safety screening of donors was known to be a critical issue in FMT before the general acceptance of the procedure by the FDA.^9,10^

## Pathogenesis of rCDI

The critical elements in the pathogenesis of CDI and rCDI are briefly outlined in Table 1 which identify therapeutic targets used to assess efficacy of FMT and LBPs.

**Table 1.** General mechanisms of pathogenesis of *Clostridioides difficile* infection that represent targets of microbiota restoration therapy.

Pathogenesis of rCDI		
Antibiotic classes differ in their potential to deplete the gut microbiome, predisposing patients to CDI and recurrences of CDI^11,12^ and producing immune and inflammatory events.^13^	Primary bile acids (e.g. cholic acid) produced in the liver lead to the germination of *C. difficile* spores^14^ with enhanced toxigenicity,^15^ with reduced levels of secondary bile acids^16^ caused by antibiotic-induced alterations of the microbiome (dysbiosis), all contributing to the pathogenesis of rCDI.	
Microbiome changes in microbiota replacement therapy of *C. difficile* recurrence by FMT or LBP		
Reestablishment of a diverse microbiome depleted by antibiotics as measured by α- and β-diversity^17^ leads to resistance to CDI and a healthy gut.^18^	Intestinal colonization resistance improves enhancing the immune system and a healthy epithelial barrier.^19^	Diverse colonic microbiota modify primary bile aids producing secondary bile acids (related to lithocholic acid), preventing *C. difficile* spore germination and growth of vegetative cells.^20^

CDI, *Clostridium difficile* infection; α-diversity, mean species diversity; β-diversity, species diversity between two microbial communities; LBP, live biotherapeutic product; rCDI, recurrent CDI.

## Available programs for microbiologic treatment of rCDI

### OpenBiome

OpenBiome, a 501(c)(3) not-for-profit organization established at Massachusetts Institute of Technology in 2012, later located in Cambridge Massachusetts, developed a stool bank to stimulate research on the human microbiome and to focus on the unmet need for prevention of rCDI in the United States. Since its beginning, OpenBiome has supervised more than 68,000 FMTs to prevent rCDI at more than 1200 hospitals and medical clinics in the United States. Chen *et al.*^21^ described the activity of OpenBiome during 2018, just before the COVID-19 pandemic. During 2018, OpenBiome received 7536 stool donations from 210 donors, a daily average of 21 donations, and processed 4271 of the fecal donations into FMT products. The median time an enrolled donor actively donated to the program was 5.8 months and the median time from manufacture of FMT preparation until shipment to a hospital or physician was 8.9 months. Thirty-six percent of donations were not accepted because of weight, form, or delay in safety testing. A complete safety examination of donors was performed based on knowledge available in 2018.

In 2022, OpenBiome developed a collaboration with the University of Minnesota Microbiota Therapeutics Program, working with Alexander Khoruts, MD, and Michael Sadowsky, PhD, from where future fecal samples will be sent for patient treatment in OpenBiome-identified centers. OpenBiome has indicated that they previously produced the product in Massachusetts and now produce it in Minnesota using Good Manufacturing Practices defined by the FDA.^21^ OpenBiome currently has an IND with the FDA for their stool bank. They provide two different FMT products for its more than 800 current treatment centers, TP-101LR which contains ⩾5 × 10^10^ bacteria in 10% glycerol and MTP-101LF which contains ⩾2.5 × 10^12^ bacteria in 10% glycerol.^22^ TP-101LR can be delivered to patients by colonoscopy/sigmoidoscopy or by naso-enteric/gastric tube for routine cases of rCDI. For fulminant CDI, TP-101LF is delivered only by colonoscopy/sigmoidoscopy. Neither product is FDA approved, indeed there is little data on the use of FMT or LBPs in fulminant CDI. Both products are stored at −20°C or colder and must be used within 6 h of thawing. The OpenBiome products cost $1695 for either preparation plus $150 for shipping.

For OpenBiome and the few research programs with independent FMT programs for treatment of rCDI with INDs with the FDA, in addition to thorough screening of donors for all known transmissible agents, they must perform potency assays of all product prepared and administered (usually a cutoff number of viable anaerobic bacteria in quantitative culture) and maintain product availability for a limited and defined period of time.

## The American Gastroenterology Association FMT National Registry

In August 2016, the American Gastroenterology Association (AGA) established an FMT National Registry with the support of the National Institutes of Health (NIH R24AI118629) with plans to enroll 4000 patients with recurrent CDI at 75 locations, following subjects for 5–10 years to determine long-term consequences of FMT.^23^ Qualified patients can learn about sites for treatment through this program through Clinicaltrials.gov and AGA FMT National Registry website.

## The modern era of LBPs

The most significant development in the harnessing of fecal bacteria to reverse dysbiosis of CDI and rCDI has occurred with the licensure of two fecal-derived LBP by the FDA, fecal microbiota, live-jslm on 30 November 2022, and fecal microbiota spores, live-brpk on 26 April 2023.

The FDA has designated LBP as ‘a biological product that: contains live organisms, such as bacteria; is applicable to the prevention, treatment, or cure of a disease/condition of human beings; and is not a vaccine’.^24^

### RBX2660 (REBYOTA™), fecal microbiota, live-jslm

Fecal microbiota, live-jslm was evaluated in three phase II and two phase III clinical trials.^25–30^

#### Description of product and instructions for administration

Fecal microbiota, live-jslm is a filtered and cryopreserved consortium of live microbiota obtained from human stool samples after the safety screening of the donors at initial assessment and each subsequent donation. Stool donations are collected at the manufacturing site and maintained at −80°C until transportation. Fecal microbiota, live-jslm products are screened as per the FDA guidance for 29 pathogens. Each dose contains between 1 × 10^8^ and 5 × 10^10^ colony forming units (CFUs) of fecal microbes including >1 × 10^5^ CFU/mL of strains of *Bacteroides* suspended in not greater than 5.97 g of polyethylene glycol (PEG3350) in 0.9% saline. R Fecal microbiota, live-jslm comes in a single dose of 150 mL, a sealed opaque vinyl acetate bag, a rectal tube, a spike port adaptor, and a clamp. The product is maintained at −60°C to −90°C, or temporarily in a standard refrigerator at 2–8°C for up to 5 days after thawing before being administered to adults 18 years of age or older, 24–72 h after receiving the last dose of anti-CDI antibiotics.

Fecal microbiota, live-jslm is administered by enema after placing the patient either in the left-side or knee-chest position illustrated in the package insert that comes with the product.^31^ The product can be administered by any trained healthcare provider in a single dose in approximately 5 min.

#### Clinical studies for safety and efficacy

Safety of fecal microbiota, live-jslm was determined in two phase III randomized, double-blind clinical trials and a phase III open-label study.^29^ All trials included 6 months of follow-up in more than 1100 subjects. The most commonly reported side effects reported in ⩾3% were abdominal pain (9%), diarrhea (7%), abdominal distension (4%), flatulence (3%), and nausea (3%).^31^ The open-labeled trials of fecal microbiota, live-jslm provided evidence of safety in 233 patients with between 6 and 12 months of follow-up and between 12 and 24 months during an exit interview.^29^

Clinical response was evaluated using a Bayesian analysis of data from two randomized, double-blind, placebo-controlled trials where treatment success was defined as the absence of rCDI, within 8 weeks of blinded treatment. In study 1^28^ of 262 adults with rCDI diarrhea, 177 subjects were randomized to fecal microbiota, live-jslm and 85 to placebo. In the second study,^30^ 39 adults received fecal microbiota, live-jslm, and 43 subjects received placebo. Clinical response was defined as no recurrence of CDI during the 8 weeks post-treatment, which was seen in 71% randomized to the fecal microbiota, live-jslm-treated groups compared with a cure rate in the placebo groups of 58%.^31^ Fecal microbiota, live-jslm was given to a sufficient number of subjects 65 years of age or older with comorbidity determined by the Charlson comorbidity index to determine safety and efficacy in this group.^32^

The durability of safety and clinical response of fecal microbiota, live-jslm, which correlated with microbiome recovery in treated subjects, was demonstrated for 2 years in 88/97 (91%) of subjects. Moreover, a stable diverse microbiome in treated subjects persisted for 8 weeks of observation. In a study of clinical response and cost, fecal microbiota, live-jslm was found to be cost-effective with $13,727 gained per quality-adjusted life year considering the cost of the drug and the prevention of hospitalization and medical management.^33^ In the second analysis, fecal microbiota, live-jslm was shown to improve health-related quality of life (HRQOL).^34^

#### Mechanisms of action

In one of the randomized placebo-controlled studies with fecal microbiota, live-jslm, stool specimens from trial subjects were examined for bile acid composition by liquid chromatography and mass spectrometry.^35^ Total bile acids were elevated at baseline and moderately reduced at the end of the trial. Primary bile acids dominated at baseline with levels falling as secondary bile acid levels increased after the LBP. The reduced fecal levels of primary bile acids and increased levels of secondary bile acids correlated with clinical response and persisted for 24 months of study.

In a phase II, placebo-controlled, double-blind trial of fecal microbiota, live-jslm treatment of rCDI, demographic comparisons between active treatment and placebo were comparable, and 16S rRNA sequencing methods were employed to characterize the fecal microbiome in 58 of 69 enrolled subjects who provided stools for examination.^25^ In clinical responders given fecal microbiota, live-jslm, α-diversity determined by Shannon index and Simpson index showed increases after treatment reaching levels seen with the administered product. β-Diversity analysis using Bray–Curtis dissimilarity studies found that the participants *versus* product were dissimilar at baseline but after treatment became more similar showing engraftment of product strains. The most prevalent phyla in the baseline samples of subjects randomized to fecal microbiota, live-jslm were Gammaproteobacteria and Bacillota with post-treatment fecal samples showing a predominance of *Bacteroides* and *Clostridium* spp. The change in microbiome composition in the group receiving fecal microbiota, live-jslm was performed using Cramer criteria,^36^ showing large differences at baseline, that increasingly approximated the composition of the product at 10, 30, and 60 days post-treatment. In placebo-treated subjects, the microbiome did not significantly change after treatment compared with baseline values. When the microbiome was studied over time using DMRepeat methods, an increase in Bacteroidetes and a decrease in Gammaproteobacteria became obvious by 10 days.

In the PUNCH CD III compared to placebo, fecal microbiota, live-jslm demonstrated a significantly higher treatment success in preventing further rCDI and enhanced HRQOL among patients at first recurrence. Fecal microbiota, live-jslm was associated with significantly higher *C. difficile* Quality of Life Survey (CDiff32) [change score difference 13.5 (standard deviation 5.7), *p* < 0.05] and mental domain [16.2 (6.0), *p* < 0.01] scores *versus* placebo from baseline to week 8.

A review summarizing all clinical studies with fecal microbiota, live-jslm has been published.^29^

### SER-109 (VOWST™), fecal microbiota spores, live-brpk

#### Description of product and instructions for administration

Fecal microbiota spores, live-brpk is a mixture of spores from fecal samples of healthy subjects consisting of approximately 50 species of non-*C. difficile* Firmicutes spores in a concentration of 1 × 10^6^–3 × 10^7^ spore CFUs in 92 ± 4% glycerol in saline. As with fecal microbiota, live-jslm, fecal microbiota spores, live-brpk product is screened for 29 pathogens. Prior to taking the first fecal microbiota spores, live-brpk tablets, the patient should have completed anti-*C. difficile* antibiotics and have a washout period of 2–4 days. The day before treatment (or at least 8 h before fecal microbiota spores, live-brpk treatment) subjects ingest 10 oz of magnesium citrate, or in the case of renal impairment 250 mL of polyethylene glycol electrolyte solution (GoLYTELY). The purgative is given with the idea of cleansing the bowel and neutralizing the residual antibiotics. Four capsules of fecal microbiota spores, live-brpk are taken each day for three consecutive days (12 total capsules) before the first meal of the day, or after at least 8 h of fasting, during which time only small amounts of water are allowed.

#### Clinical studies for safety and efficacy

The safety of fecal microbiota spores, live-brpk was evaluated in one phase III, randomized, double-blind clinical study^37^ and one open-label clinical study.^38^ The common adverse reactions to fecal microbiota spores, live-brpk (*versus* placebo), occurring in ⩾5% of subjects were abdominal distension 31% (*versus* 29%), fatigue 22% (*versus* 22%), constipation 14% (*versus* 11%), chills 11% (*versus* 8%), and diarrhea 10% (*versus* 4%).^39^ In study 1, the reactions occurred within 10 days of starting treatment and declined during follow-up. The majority were mild or moderate in severity. Within the fecal microbiota spores, a live-brpk-treated population of 349 subjects, there were no serious events related to the study drug as determined by the investigators. There are no data on the use of fecal microbiota spores, live-brpk in pregnancy or children. Of the 349 subjects treated with fecal microbiota spores, live-brpk, 183 (52%) were aged 65 and older. There were insufficient data to determine whether elderly subjects responded differently than younger adults.

Four clinical trials have been carried out to evaluate the efficacy of fecal microbiota spores, live-brpk in treating rCDI, ‘phase I study and ECOSPOR II, III, and IV’.^40^ While an initial phase Ib trial gave fecal microbiota spores, live-brpk in reducing rCDI failed to demonstrate the efficacy of fecal microbiota spores, live-brpk over placebo.^41^ The last two trials ECOSPOR-III and ECOSPOR-IV met the targets for efficacy for fecal microbiota spores, live-brpk in preventing post-treatment CDI. In these later trials to boost efficacy, the drug dose was changed from a single dose to four capsules daily for three consecutive days and the spore count was increased to 3 × 10^7^ CFU.^37,41^ ECOSPOR-IV trial, an open-label extension and an open-label study, demonstrated sustained clinical response (cure) through week 8 (91%) and week 24 (95%).^40^ The efficacy of fecal microbiota spores, live-brpk was evaluated in a dose of four capsules daily for 3 days for rCDI in a randomized placebo-controlled multi-center study (ECOSPOR III).^37^ In an intent-to-treat analysis of 182 subjects, 11 of 89 (12%) in the fecal microbiota spores, the live-brpk-treated group developed CDI within 8 weeks post-treatment, compared with 37 of 93 (40%) subjects randomized to placebo (*p* < 0.001). In a secondary analysis of this phase III clinical study, an exploratory analysis of HRQOL was performed revealing a consistent improvement in in HRQOL over time.^42^

#### Mechanism of action

Engraftment of species from the product, fecal microbiota spores, live-brpk was seen in stool samples of treated subjects beginning within the first week of treatment with the number of species that engrafted rising after this time point and remaining high through week 8.^37^ In the second study, fecal microbiota spores, live-brpk led to microbiome repair and provided a sustained response up to 24 weeks post-therapy.^38^ This spore product was shown to reduce proinflammatory Proteobacteria and facilitate the growth of Firmicutes including families of Ruminococcaceae and Lachnospiraceae^37^ and to increase fecal secondary bile acids.^37,41^

A review summarizing all clinical studies with fecal microbiota spores, live-brpk has been published.^40^

In Table 2, the various LBPs available are compared. Also, included in the table are available options for FMT.

**Table 2.** Comparison of the various forms of bacteriotherapy available, fecal microbiota transplantation, and newly licensed live biotherapeutic products.

Product type, company, name	How to access	Pre-treatment bowel prep needed	Treatment type and regimen	Product characterization pre-treatment	Cost	Efficacy^a^ in published studies
Investigational FMT OpenBiome FMT (two concentrations)	Find a physician in a zip code by searching OpenBiome.org/find-a-doctor	Bowel prep for colonoscopy/sigmoidoscopy administration	Solution: One infusion by colonoscopy/sigmoidoscopy or by nasogastric tube	No	$1695 for either preparation plus $150 for shipping	In 267 patients blinded to treatment, cure was seen in 70.4% given RBX2660 and 58.1% in placebo (posterior probability value of superiority of 0.991).^28^
Investigational FMT OpenBiome through the AGA FMT	By application through https://gastro.org/research-and-awards/registries-and-studies/fecal-microbiota-transplantation-national-registry/	Bowel prep for colonoscopy/sigmoidoscopy administration	Solution: One infusion by colonoscopy/sigmoidoscopy or by nasogastric tube	No	No cost for product, only administration should be covered by insurance	
FDA-approved (licensed) treatment LBP Ferring Pharmaceuticals RBX2660 (Rebyota™) generic name, fecal microbiota, live-jslm	Prescription from a physician	None	Solution: One enema of 150 mL of product	Yes	$9000 Covered by most insurance plans	Clinical response was defined as no recurrence of CDI during the 8 weeks post-treatment, which was seen in 71% randomized to the fecal microbiota, live-jslm-treated groups compared with a cure rate in the placebo groups of 58%.^31^
FDA-approved (licensed) treatment LBT Seres Pharmaceuticals and Nestle SER-109 (VOWST), generic name fecal microbiota spores, live-brpk	Prescription from a physician	Magnesium citrate 10 oz the day before	Capsules: four capsules/day for 3 days on an empty stomach	Yes	$17,500 Covered by most insurance plans	In an intent-to-treat analysis of 182 subjects with rCDI, 78/89 (88%) randomized to SER-109 were protected from subsequent bouts of CDI compared with 56/93 (60%) randomized to placebo (*p* < 0.001).^37^

a Rate of prevention of rCDI in treated patients in a placebo-controlled trial; note that the methods differed in the various LBP trials.

AGA, American Gastroenterological Association; FDA, Food and Drug Administration; FMT, fecal microbiota transplantation; rCDI, recurrent *Clostridioides difficile* infection, LBT, live biotherapeutic products.

## Discussion

A microbiota approach to prevention of recurrent CDI is the most effective treatment for preventing recurrences as shown by a comparative trial of FMT *versus* fidaxomicin in rCDI,^43^ and by a systematic review and meta-analysis of available studies.^44^ A European Consensus Conference on FMT in clinical practice viewed the quality of evidence as high and the strength of evidence strong for the use of FMT for rCDI.^45^

The licensure and availability of two safe and effective standardized and regulated biotherapies, fecal microbiota, live-jslm and fecal microbiota spores, live-brpk, for preventing rCDI is a critical advance in medical management. Both treatments were shown to cure rCDI, to normalize the microbiome of the treated patients by reducing proportions of proinflammatory Enterobacteriaceae and increasing the α- and β-diversity of the microbiome, and to convert primary bile acids to *C. difficile*-inhibiting secondary bile acids in fecal samples. Both products included follow-up studies to show that the therapeutic agents lead to a cure of the infection without important short-term adverse events, although fecal microbiota, live-jslm studied patients out to 2 years.^46^ The clinical efficacy of fecal microbiota, live-jslm and fecal microbiota spores, live-brpk in a number of cited studies is comparable with results seen with FMT,^21^ although head-to-head trials are needed to be certain. In studies with lower than expected clinical response, a suboptimal dose of the study drug was used or a greater placebo response than expected was seen.

While the two recently licensed LBP are both derived from fecal samples of healthy well-screened donors, they differ in a number of ways. Fecal microbiota, live-jslm is a broad consortium of microbiota expected in healthy donor fecal samples, with all the major phyla including Firmicutes. It is augmented with strains of Bacteroidetes, while fecal microbiota spores, live-brpk is ethanol-washed spores exclusively within the phylum of Firmicutes. The fact that both products are effective in preventing rCDI supports the idea that bacterial restoration in rCDI can be achieved by transplantation of a variety of different microbiota. This is seen in FMT for rCDI where it is generally accepted that all healthy adults not recently receiving antibiotics are suitable donors and a large number of donors can be included unscreened for microbiome diversity in a stool bank such as OpenBiome. When treating conditions other than CDI, the specific makeup of an LBP may need to be adjusted.^47^ One way around the unique microbiome requirements of non-CDI illnesses with dysbiosis is to administer FMT products derived from multiple donors.^48^

Evidence developed and presented here indicates that the two new LBPs show similar rates of efficacy in treating rCDI, although head-to-head comparisons have not been carried out. Fecal microbiota, live-jslm is a more traditional microbiome restoration product employing a full range of microbiota including strains of Firmicutes and Bacteroides so is likely to be effective in rCDI caused by a wide variety of strains of *C. difficile* strains with varying degrees of dysbiosis and different patterns of microbiota alteration. Fecal microbiota spores, live-brpk is novel in design and is based on the selection of Firmicutes spores with a narrower range of bioactivity. It is of interest that fecal microbiota spores, live-brpk consisting of microbiota spores from only one phylum, appear appropriate for the treatment of rCDI. Butyrate-producing strains of Firmicutes may be critical to replete the dysbiosis in rCDI.^49^ This fits with our studies with a lyophilized FMT product given orally to patients with rCDI where engraftment of Firmicutes correlated best with recovery from rCDI.^17^

The future of microbiota-therapy has gotten brighter with the licensure of fecal microbiota, live-jslm and fecal microbiota spores, live-brpk. While these products provide an effective treatment for rCDI, they are first-generation LBPs that need to be evaluated in other conditions associated with dysbiosis.^50^

For patients unable to obtain one of the new LBPs, FMT is currently available through OpenBiome. The AGA FMT National Registry project in which patients treated with FMT will be followed for up to 10 years will be invaluable in confirming the long-term safety of administering fecal-derived products to humans.

In the future, we hope that LBPs will employ non-fecal-derived microbiota that will be easier and less expensive to produce and in pure form with fewer potential adverse events. We await clinical evidence to support this concept. Meanwhile, this is a great beginning.

## Declarations

**Ethics approval and consent to participate:** Not applicable.

**Consent for publication:** Not applicable.

**Availability of data and materials:** Not applicable.
